# The elusive tau molecular structures: can we translate the recent breakthroughs into new targets for intervention?

**DOI:** 10.1186/s40478-019-0682-x

**Published:** 2019-03-01

**Authors:** Yann Fichou, Youssra K. Al-Hilaly, François Devred, Caroline Smet-Nocca, Philipp O. Tsvetkov, Joke Verelst, Joris Winderickx, Nick Geukens, Eugeen Vanmechelen, Audrey Perrotin, Louise Serpell, Bernard J Hanseeuw, Miguel Medina, Luc Buée, Isabelle Landrieu

**Affiliations:** 1 Department of chemistry and biochemistry, University of California Santa Barbara, Santa Barbara, CA 93106 USA; 2 Dementia Research group, School of Life Sciences, University of Sussex, Falmer, E Sussex, BN1 9QG UK; 3 Chemistry Department, Science College, Mustansiriyah University, Baghdad, Iraq; 4 Aix-Marseille University, CNRS, INP, Inst Neurophysiopathol, Fac Pharm, Marseille, France; 5 University of Lille, CNRS, UMR8576, 59000 Lille, France; 6 Functional Biology, KU Leuven, Kasteelpark Arenberg 31, Box 2433, 3001 Heverlee, Belgium; 7 Pharmabs, KU Leuven, Herestraat 49, box 820, B 3000 Leuven, Belgium; 8 ADx NeuroSciences NV, Technologiepark Zwijnaarde 94, 9052 Ghent, Belgium; 9 Life Molecular Imaging, Berlin, Germany; 10 Neurology Department, Cliniques Universitaires Saint-Luc, 10 Avenue Hippocrate, 1200 Brussels, Belgium; 11 Gordon Center for Medical Imaging, Radiology Department, Massachusetts General Hospital, Harvard Medical School, Boston, MA USA; 12 CIBERNED (Network Center for Biomedical Research in Neurodegenerative Diseases), Madrid, Spain; 13 CIEN Foundation, Queen Sofia Foundation Alzheimer Center, Madrid, Spain; 14 University of Lille, Inserm, CHU-Lille, UMRS1172, 59000 Lille, France

**Keywords:** Tauopathies, Alzheimer’s disease; tau structure; tau aggregation, Alzheimer’s disease diagnosis, Amyloid

## Abstract

Insights into tau molecular structures have advanced significantly in recent years. This field has been the subject of recent breakthroughs, including the first cryo-electron microscopy structures of tau filaments from Alzheimer’s and Pick’s disease inclusions, as well as the structure of the repeat regions of tau bound to microtubules. Tau structure covers various species as the tau protein itself takes many forms. We will here address a range of studies that help to define the many facets of tau protein structures and how they translate into pathogenic forms. New results shed light on previous data that need now to be revisited in order to up-date our knowledge of tau molecular structure. Finally, we explore how these data can contribute the important medical aspects of this research - diagnosis and therapeutics.

## Introduction

**Fig. 1:**
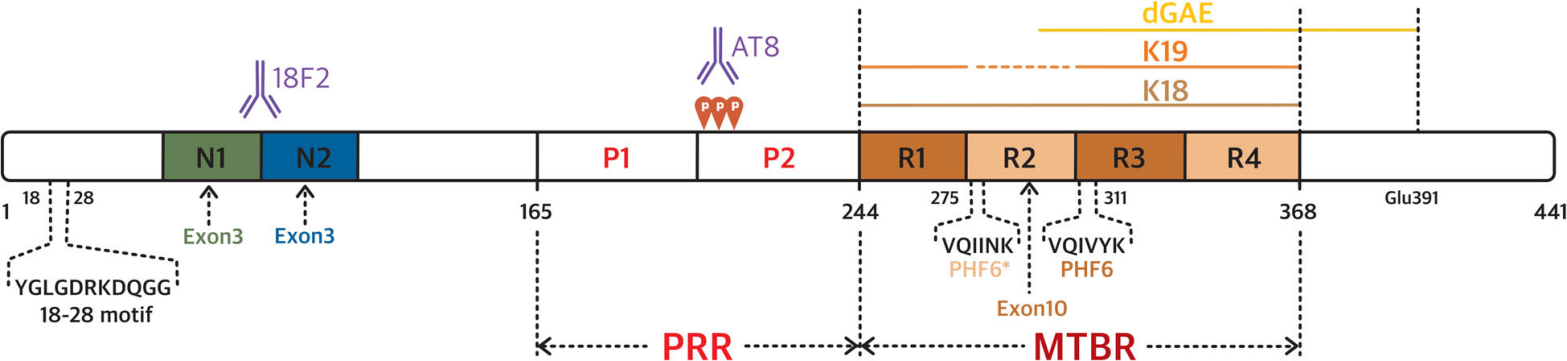
Scheme of tau showing domain organization. Depending of the isoform, tau has an N-terminal extension with 0, 1, or 2 inserts (tau0N, tau1N, tau2N, respectively), the presence of N1 and N2 inserts depending on exon 2 and exon 3, respectively. The microtubule-binding region (MTBR) has three (tau3R) or four (tau4R) repeats, the presence of R2 depending on exon 10. MTBR repeats R1 to R4 (31 or 32 residues for each repeat and inter-repeat region) have similar sequences. The PHF6* and PHF6 peptides are located in R2 and R3, respectively. The longest tau isoform corresponds to 441 amino-acid residues (or tau2N4R) and the shortest to tau352 amino-acid residues (or tau0N3R). Tau fragments K18, K19 and dGAE are mentioned in the text. The proline-rich region or PRR has many phosphorylation sites, combination of pS202/pT205 and pS208 forms the AT8 monoclonal antibody epitope. Antibody 18F12 recognizes a conformational epitope at the junction of N1 and N2 inserts. The 18–28 motif of tau is primate specific

The investigation of tau molecular structure covers its primary sequence, local elements of secondary structure and global fold and, finally, complex formation and aggregation. Tau is defined as an intrinsically disordered protein (IDP) and is present as six isoforms in human brain (ranging from 352 to 441 amino acid residues, Fig. 1), resulting from alternative splicing [53, 63]. All these isoforms are themselves subjected to multiple post-translational modifications (PTMs), the best studied being phosphorylation. Its PTMs are incredibly complex because they are numerous and can combine in many ways [105] and these are also prone to cross-talk [20, 85, 86]. To make matters even more complex, tau proteins are subjected to proteolytic degradation [33, 171]. Ratios of isoforms [37], level of phosphorylation and proteolytic degradation all contribute to normal and pathological tau activity. Tau has many binding partners, and is most notoriously bound to microtubules (MTs) [49, 73, 76, 93, 150, 162, 167], but also to many regulatory proteins [90, 115], DNA [17, 122] or membrane [2]. The later non-standard functions of tau were previously reviewed following EuroTau 2017 gathering [141].

Tau is associated with several neurodegenerative diseases, termed tauopathies, in which it is found as insoluble deposits associated with other cellular components [13]. Tau is the principal component of paired helical filaments (PHFs) and straight filaments (SFs) [23, 54], which form the intraneuronal fibrillar deposits known as neurofibrillary tangles (NFT) in Alzheimer’s disease (AD) and leading ultimately to neurofibrillary degeneration. The tau filaments have cross-β structure and therefore share the common characteristics of amyloid [18]. The aggregates themselves are not a single homogenous family. In addition to mature insoluble aggregates, which have different disease-specific structures, tau is found as oligomeric forms, loosely defined, but thought to have a very toxic effect [42, 62]. Thus, inhibition and/or clearance of oligomeric hyperphosphorylated tau could be a more effective therapeutic target than the fibrilized protein [82, 143]. Because tau filaments were thought to be composed of the microtubule binding region (MTBR, Fig. 1), it has been common practice to use tau fragments containing only this region, either in its 3R (isoform containing 3 repeat domains) version (K19) or 4R (isoform containing 4 repeat domains) version (K18), as model peptides for aggregation studies. Two homologous hexapeptides named PHF6* (_275_VQIINK_280_) and PHF6 (_306_VQIVYK_311_) located at the start of the second and third repeat regions (R2 and R3) (Fig. 1) of tau MTBR, respectively, are essential for tau aggregation [157]. PHF6* is thought to be the stronger driver of aggregation [135]. PHF6(*) (PHF6* and PHF6) peptides spontaneously aggregate in solution in contrast to the full-length tau which is a highly soluble protein. The atomic structures of the two hexapeptides reveal the capacity of these segments to form interdigitated steric-zipper interfaces that seed tau aggregation [79, 131, 135].

To grasp the molecular characteristics of tau structures is challenging. First of all, as a large IDP, tau is flexible and dynamic and requires high-field nuclear magnetic resonance spectroscopy (NMR) to collect molecular detail. Tau has a low complexity amino-acid sequence, and recently joined the club of proteins with the capacity to form liquid droplets [8]. More than an oddity, it seems that this form of tau is able to seed MTs assembly in a highly efficient manner and might have consequences for aggregation initiation [8, 161]. Aggregates are solid and heterogeneous, and therefore are challenging to characterize by classical structural techniques. Finally, the molecular details of tau interaction with MTs are difficult to define due to the dynamic nature of the complex, the MTs being by themselves in dynamic equilibrium. Progress in understanding the mechanistic role of tau as a microtubule associated protein came from cryo-electron microscopy (cryo-EM), which offered a view of tau repeats bound to MTs [76]. Recent breakthroughs, detailed in this review, came from progress in sophisticated biophysical techniques brought together with immense efforts and ingenuity.

We here will focus on tau molecular structures, highlighting the techniques required for its characterization, and summarizing the results that may provide basis for a better definition of tau pathological forms and the pathway(s) of pathogenesis. Finally, we conclude by showing how these results can translate into better targeted tau-antibodies for diagnostic and into progress in tau imaging. This review does not intend to be a full-coverage of the literature but rather to reflect the lively discussion that took place on the EuroTau meeting 2018, in Lille, France.

### Aggregate structure: From heparin-induced structure to native conformation

The characterization of amyloid structures is challenging because they are only partially ordered and often heterogeneous. Crystallization has been possible for short peptides [125, 135], but not for full-length proteins. Because of this lack of precise structural information, the relationship between amyloid structure and pathology remains a heated debate for many proteins; tau is no exception.

The large majority of structural studies in the last few decades have been carried out on aggregates made out of recombinant tau constructs. Limited proteolysis applied on K18, K19 and the full-length tau2N4R showed that the amyloid core is formed by the second half of R1, R2 (when present), R3 and the first half of R4 [156]. Solid-state NMR (ssNMR) confirmed that, in K19, β-sheets are formed in the end of R1, in the full R3 and the beginning of R4 [12]. Another ssNMR study showed more precisely that only 19 residues, 306–324, formed β-sheets while the rest remains relatively dynamic [29], in agreement with proton/deuterium exchange experiments. They also showed that the packing is in-register and parallel, confirming what was observed earlier by electron paramagnetic resonance (EPR) spectroscopy [91]. Furthermore, Bibow and co-workers [19] showed that the N- and C-termini (0–212, 399–441) are highly mobile while the central region is too immobile to be detected by solution NMR. They also show electrostatically-driven long-range interactions between the filament core and both C- and N-terminal extremity.

While recombinant filaments have shed light on many aspects of tau aggregation mechanisms and structure, it is important to note that their formation presents potential biases: (i) the use of an arbitrary cofactor, (ii) the absence of PTMs, (ii) the use of an arbitrary tau segment. Therefore, it remains today unclear how much of the atomic arrangements found in recombinant filaments is biologically relevant.

When extracting aggregates from brain, trypsin resistant cores show different pattern in gel electrophoresis for Pick’s disease (PiD), AD, progressive supranuclear palsy (PSP) and corticobasal degeneration, suggesting different core composition/structure for each disease [148].

The recent technological breakthroughs of cryo-EM have allowed to solve two structures of tau aggregates, extracted from AD- and PiD-affected human brains [40, 44]. These structures represent major advancements in the field as they provide the first high-resolution molecular architectures of the tau filament cores extracted from human tissues. The AD protofilament core is similar for PHFs and SFs and is composed of R3 and R4 repeat domains as well as 10 residues in the C terminus region (306–378), and exhibit a Greek-key conformation [44], reminiscent of the α-synuclein fibril structure [152]. The core also forms a region of β-helix similar to the conformation identified in the prion-forming domain of HET-s, in which it plays a crucial role for infectious properties [159]. The major difference between SFs and PHFs lies in the interaction between the two protofilaments. It should be noted that additional electron densities were detected in the region before R3, strongly suggesting that part of R2 (for 4R tau) or R1 (for 3R tau) is also partially structured in AD filaments. Similar structural features were found in several sporadic and inherited AD brains, suggesting that this structure is a hallmark of AD [41]. The PiD filament core encompasses AD’s (306–378) and also includes half of the R1 repeat [40]. The structure shows that the arrangement of the R1 (254–274) segment is not compatible with the equivalent sequence in R2 (285–305), providing an explanation as to why PiD aggregates contain only 3R tau isoform. Although most of the β-sheets in the region 306–354 locally align between AD and PiD, the filament core from the latter possesses neither a Greek-key shape nor a β-helix, characteristic of AD filaments. It should be noted that although cryo-EM provided two well-defined structures, it does not exclude the existence of other minor aggregate species, which would be excluded during the image analysis process.

The high-resolution structures of brain-extracted tau aggregates reveal that filaments formed by recombinant full-length tau seem to be different from in-vivo filaments. Indeed, ssNMR and limited proteolysis predicted immobile and/or protected regions (see paragraphs above) different from either AD or PiD filament core. It was notably unexpected to have a structured core extending beyond the R4 region. This finding suggests that the widely used K19 and K18 fragments, composed exactly of the 3 or 4 repeat domains, might not be able to model PiD and AD filament cores as they lack the C-terminal region 368–378. In addition, it was shown by EPR spectroscopy that heparin-induced filaments from a tau fragment 255–441 do not reproduce the large domain folds present in AD and PiD cores [43]. The authors also demonstrated that heparin filaments are highly heterogeneous. These differences between brain-extracted aggregates and heparin-induced filaments were later confirmed and further described by cryoEM [170].

The difficulty in obtaining biologically relevant structures with recombinant proteins could in principle be overcome by seeding aggregation using tissue-extracted material. Seeding refers to the process by which monomers are recruited by premade aggregate, therefore multiplying the quantity of aggregate, either in vitro or in vivo. Although it is generally thought that seeding faithfully propagates the structure of the seed, this assumption has not yet clearly been demonstrated at high resolution. On the one hand, cellular seeding assays have showed that the macroscopic aspects as well as the patterns of limited proteolysis of a given strain can be maintained through several seeding generations [75], suggesting the propagation of the seed structure. On the other hand, different tau constructs (tau2N4R, K18 and K19) have been shown to form different limited-proteolysis signature, which cannot be propagated for more than one generation of in-vitro seeding [111]. The authors suggested that the seeded protein, and not the seed, determine the pattern of the final filament. EPR spectroscopy has been used to characterize the population of aggregates at high resolution by measuring intramolecular distance distributions inside the filament core. Meyer and coworkers showed that filaments species are selected through seeding cycles depending on their mechanical properties and the environmental conditions [100]. The same group highlighted a cross-seeding barrier between different fragments (in particular K18 cannot seed K19 while K19 can seed K18) that they attributed to conformational incompatibility between the seed and the seeded species [138]. They furthermore showed that the seeded barrier can be modulated by single-point mutations in the core domain or by interactions with the N-and C-terminal tails [100, 163]. It should be noted that in-vitro seeding is often assisted by a cofactor (RNA or heparin), which was recently shown to be an essential component of the filaments as they are necessary to ensure stability of either seeded or non-seeded filaments [43]. The biological relevance of these co-factors is not well established, as there are many potential co-factor candidates in the cellular environment. Heparan-sulphate proteoglycans were reported to be associated with AD tau filaments [51], although they are not present in the intracellular medium where tau is mostly found. RNA was also shown to be sequestered in tau fibers from several tauopathies [50]. Other proteins might also be potent co-factors such as α-synuclein [106] and even nuclear pore proteins [38].

### Tau fragments and aggregation

Tau truncation plays an important role in AD pathology [113]. Truncated tau proteins were initially identified as constituents of the pronase-resistant PHF core [165, 166], and it has been suggested that tau truncation drives the pathological conversion of wild-type tau at neuritic plaques [83]. Caspase activation associated with tau truncation led to tau aggregation in tau transgenic mice and expression of a tau fragment cleaved at Asp421 (mimicking caspase truncation) into wild-type mice led to the appearance of intracellular aggregates [32]. Intriguingly, gingipain proteases secreted by *Porphyromonas gingivalis*, a pathogen involved in chronic periodontitis and able to invade the brain, have been proposed to affect tau by direct gingipain proteolysis as well as gingipain activation of human proteases that act on tau [36].

In vitro, C-terminal truncated tau at Glu391 or at Asp421 have a higher propensity to aggregate than full-length tau when using an aggregation inducer [1, 45, 168]. A truncated form of tau has been identified in AD brain tissue which extends from residue 297 to residue 391, known as dGAE [70, 166]. The antibody mAb423 can be used to identify the specific fragments which terminate at Glu391 [60], and has been shown to bind to intra- and extracellular NFTs in AD brain tissue [98, 112, 139]. The presence of this truncated tau form was confirmed using MS analysis for pronase-untreated PHFs extracted form AD brain (Braak stage V: [21]). This resolved the long running debate as to whether tau truncation is a result of pronase treatment, or whether it occurs naturally in disease [173].

Polyanions such as heparin have been widely used to produce tau aggregates for in vitro studies [15, 18, 51, 157]. However, recent work using circular dichroïsm (CD) revealed that heparin interacts directly with a drug that was produced by TauRx Therapeutics called leuco-methylthioninium [3], thus developing a new heparin-free in vitro model became essential. In addition, heparin-induced filaments (see above) were found structurally different from those in AD brain [43].

**Fig. 2:**
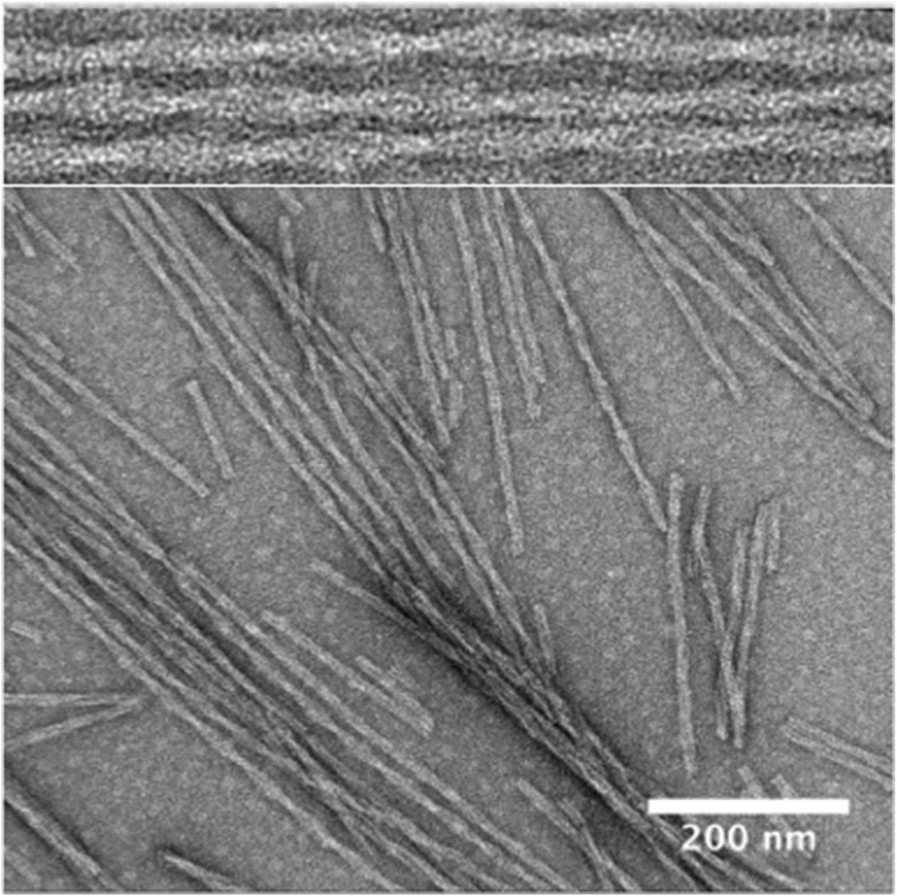
Negative stain EM image of in vitro PHFs produced from dGAE in additive-free conditions at pH 7.4 and 37 °C [4]

A new in vitro model system was developed to produce aggregates from the truncated PHF-core tau fragment, dGAE, using physiological conditions and without any additives [3, 4]. The resulting filaments closely resembled PHFs found in AD brain (Fig. 2), sharing a similar periodicity of 65–80 nm [4, 142]. Furthermore, the preparation produces a subset (about 10%) of SF-like filaments, similar to the ratio found from tissue extraction [44]. dGAE includes the PHF core region identified in the recently reported PHF structure [41, 44]: residues 323–335 of R3 and 354–369 of R4. Thus, this in vitro model will help researchers in the field to better understand the tau misfolding process into PHFs and SFs and the molecular mechanism of tau propagation.

The role of disulphide links in tau assembly into PHF remains still unclear in full-length tau and in various truncated tau fragments [66]. Full length tau in COS cells (fibroblasts from monkey kidney tissue) has been reported to form two types of dimer, one cysteine-dependent and the other, cysteine-independent [129]. Similarly, dGAE is able to form both dimer types [4]. Variants of full-length tau (C291A/C322A) and truncated tau dGAE (C322A) have been shown to be able to form filaments [4, 129]. Importantly, the self-assembly and elongation of the filaments formed by dGAE is enhanced under reducing conditions, signifying that the cysteine found at position 322 is not required to form a disulphide bond for assembly of this truncated region [4]. This is backed by the structural details of AD isolated PHF which show Cys322 to be buried in the interior of the structure [44], in contrast with the PiD fold where it is exposed to solvent [40]. This suggests that disulphide bond formation is not favourable to form AD PHF. In addition, inhibition of tau aggregation using a small molecule, leuco-methylthioninium, was found to be cysteine-independent [3], confirming that disulphide bond formation is not necessary for tau assembly.

### Tau phosphorylation and aggregation

Tau phosphorylation is physiological and regulates, among many interactions, the tubulin polymerization capacity of tau, to maintain the dynamic character of MTs in normal physiological conditions, while tau protein found in PHF is hyperphosphorylated [55]. In pathological conditions, the aggregation of tau is commonly described as being a consequence of tau hyperphosphorylation [5, 158]. The initial characterization was performed by mass spectrometry (MS) on PHFs isolated from patients and showed an average of 20 modified phosphorylation sites compared to an estimated four to five for the soluble protein [56, 62, 103]. In addition, in vitro tau aggregation assays performed by addition of polyanionic molecules, such as heparin, is proposed to mimic the seeding effect of phosphorylated tau on the unphosphorylated protein [6]. Finally, phosphorylated tau, extracted from brain of AD affected patients, or in vitro phosphorylated by rat brain extract, was shown to be more sensitive to aggregation in in vitro assays, compared to the unmodified wild-type protein [5]. From these early results, an attractive model of the impact of phosphorylation on tau pathogenesis was proposed: hyperphosphorylated tau would detach from the MTs and aggregate, phosphorylation initiating both aspects.

Although quite seductive, this model does not recapitulate all recent results, and although it has its merits, it is probably still a simplified view of the impact of tau phosphorylation on its aggregation propensity. Indeed, hyperphosphorylated tau, obtained by in vitro phosphorylation with recombinant ERK kinase (15 phosphorylated sites), rat brain extract (18 phosphorylated sites) [123] or obtained from recombinant expression in insect cells (10 or 20 phosphorylation sites*)* is not significantly more susceptible to in vitro aggregation than its wild-type counterpart [149]. In the case of ERK-phosphorylated tau, a few filaments are observed by EM from the pellet of the aggregation assay. However, the aggregation is not detected by the classical Thioflavin T fluorescence (ThT) associated with β-sheet aggregate formation in in vitro assay, indicating that the filaments are a minor fraction of tau in the sample [123]. Accordingly, tau which was highly phosphorylated by recombinant expression in insect cells shows increased oligomerization but not tau fibrillization per se [149]*.* The observation that in vitro aggregation propensity of the in vitro hyperphosphorylated tau is low does not exclude that it could be a trigger in a cellular context. Indeed, in cellular context additional elements are in play [42] some depending on Tau phosphorylation status, such as interaction with co-factors [50, 51], increase in local concentration due to detachment from the MTs [7] and/or deficient degradation [126], as well as Tau proteolysis (see preceding paragraph).

In addition, not only the number of phosphorylation sites, but also phosphorylation positions should be considered, as not all phosphorylations are equivalent. Most likely a specific combination of phosphorylation sites lies at the basis of tau becoming oligomerisation/aggregation prone [154], although the exact combination is unknown. Keeping this point in mind, a decrease phosphorylation of tau, globally or at some sites, is compatible with an increase aggregation, depending on which sites are affected.

Moreover, tau is described to misfold on its pathway of aggregation, although the definition of what is a misfolded IDP is not straightforward. Some data indicate early conformational changes that could be early stages of misfolding taking place. For example, the MC-1 or Alz50 antibodies [24] recognize conformational epitopes and detect abnormal tau in early stages of AD. Pseudophosphorylations (replacement of Ser and Thr residues by Glu residues) to reproduce the AT8 (the AT8 epitope is defined in this study as a combination of pSer199, pSer202 and pThr205), AT100 (pThr212 and pSer214), and PHF1 (pSer396 and pSer404) epitopes were used to evaluate the impact of the phosphorylation on tau global conformation based on distance measurements from FRET-pairs. A more compact global fold was found compared to the wild-type, increasing contact between the N and C-terminal regions (paper-clip fold), better reproducing the conformation recognized by the conformational antibody MC-1 that targets AD-tau [71]. A recent study based on cross-linking coupled to MS probed the structural differences between seed-competent or inert tau monomers, including tau monomers purified from AD and control brains. In these seed-competent monomers, the amyloidogenic peptides PHF6(*) were more accessible compared to inert (unable to seed aggregation) purified tau monomers from control brain [101]. Shielding the PHF6(*) sequences in the inert monomer was attributed to a preferential hairpin conformation of tau around these regions. This study was in agreement with earlier work based on EPR spectroscopy showing that exposure of tau to aggregation-promoting cofactor heparin opens up and exposes PHF6(*) regions [39]. These studies suggest a structural origin for the initiation of tau aggregation with conversion of tau monomer from an inert to an aggregation-prone form that could be viewed as an early misfolding intermediate.

In view of these data, and at the molecular level, two points should be considered to refine the concept of the impact of tau phosphorylation on its susceptibility to aggregation: 1/ the effect of specific pattern of phosphorylation and 2/ the impact of these phosphorylation events, not only on the electrostatic character of tau, but also on tau local structure and global fold.

With these points in mind, the impact of phosphorylation on Ser202 and Thr205 was investigated using NMR spectroscopy. pSer202 and pThr205 are part of the epitope for the well-known AT8 monoclonal antibody used in many studies to detect what is defined as a pathological tau protein. What was observed for the AT8-phosphorylated tau is the formation of a particular dynamic turn conformation, which is stabilized by a hydrogen bond from the phosphate of the pThr205 residue side-chain to the amide proton of Gly207. The turn conformation is further stabilized by Arg209 and Arg211 residues that face the pSer202/pThr205 residues with Gly207 located in the middle of the positively and negatively charged sequences, inducing backbone flexibility [46]. Tau protein showing this pattern of phosphorylation, in combination with absence of phosphorylation of the Ser262 residue to avoid interference, is not more sensitive to aggregation than the wild-type protein [35]. However, combined phosphorylation at the Ser202/Thr205/Ser208 sites, together with absence of phosphorylation of the Ser262 residue, yields a tau sample that forms filaments, as observed by ThT fluorescence and EM, and this triple phosphorylation state of AT8 epitope alone is sufficient to induce aggregation of tau in vitro [35]. This triple phosphorylation pattern was suggested to represent a better epitope for the AT8 monoclonal than the double Ser202/Thr205 phosphorylation [89]. The crystal structure of the complex of antibody with a pSer202/pThr205/pSer208 phosphorylated tau peptide showed no turn conformation of the bound epitope. Accordingly, in solution, no turn-like conformation was detected for the triple-phosphorylated AT8 epitope. Whether the conformation could be part of the increased susceptibility to aggregation was investigated using a mutated tau protein with Gly207 replaced by a Val residue exhibiting a bulky, Cβ-branched side chain. This mutation disrupts the formation of the dynamic turn, even in the presence of pSer202/pThr205. Interestingly, susceptibility to in vitro aggregation of a tau fragment containing the Gly207Val mutation is increased compared to the pSer202/pThr205 or unphosphorylated tau protein. These experiments show that a conformational change induced, either by a mutation (although Gly207Val mutation has never been found in any tauopathy) or a specific phosphorylation pattern, could alter the aggregation propensity of tau, and a large number of phosphorylated sites is not required to change this propensity. It might be that not only the additional charges, but also the subtle impact on tau dynamic structure is an important parameter. The molecular mechanism by which a dynamic turn located at the AT8 epitope could prevent in vitro tau aggregation (in the absence of heparin inducer) remains to be defined. It might also not be the only pattern of phosphorylation that could have this protective effect. To reconcile these in vitro data with the body of research conducted in the cellular context is not straightforward. First, the AT8 can recognize both the double pSer202/pThr205 and triple pSer202/pThr205/pSer208 phosphorylation pattern that showed differential effect on tau aggregation. Secondly, AT8 positive tau species are modulated by other phosphorylation, and, for example, phosphorylation of Ser262 prevents in vitro aggregation [133], whatever the status of the AT8 epitope (2 or 3 phosphorylations).

Nevertheless, the structural studies show a facet of the complex impact of tau phosphorylation on aggregation. Although tau is disordered, it can indeed be described as misfolded at the global and local level, due to specific phosphorylation.

### Zinc binding and tau aggregation

Zinc, the most abundant trace metal in brain, is known to play an important regulatory role both in a number of physiological processes, including neuronal growth and signal transduction, and in the pathogenesis of several neurodegenerative diseases such as AD [104, 160].

**Fig. 3:**
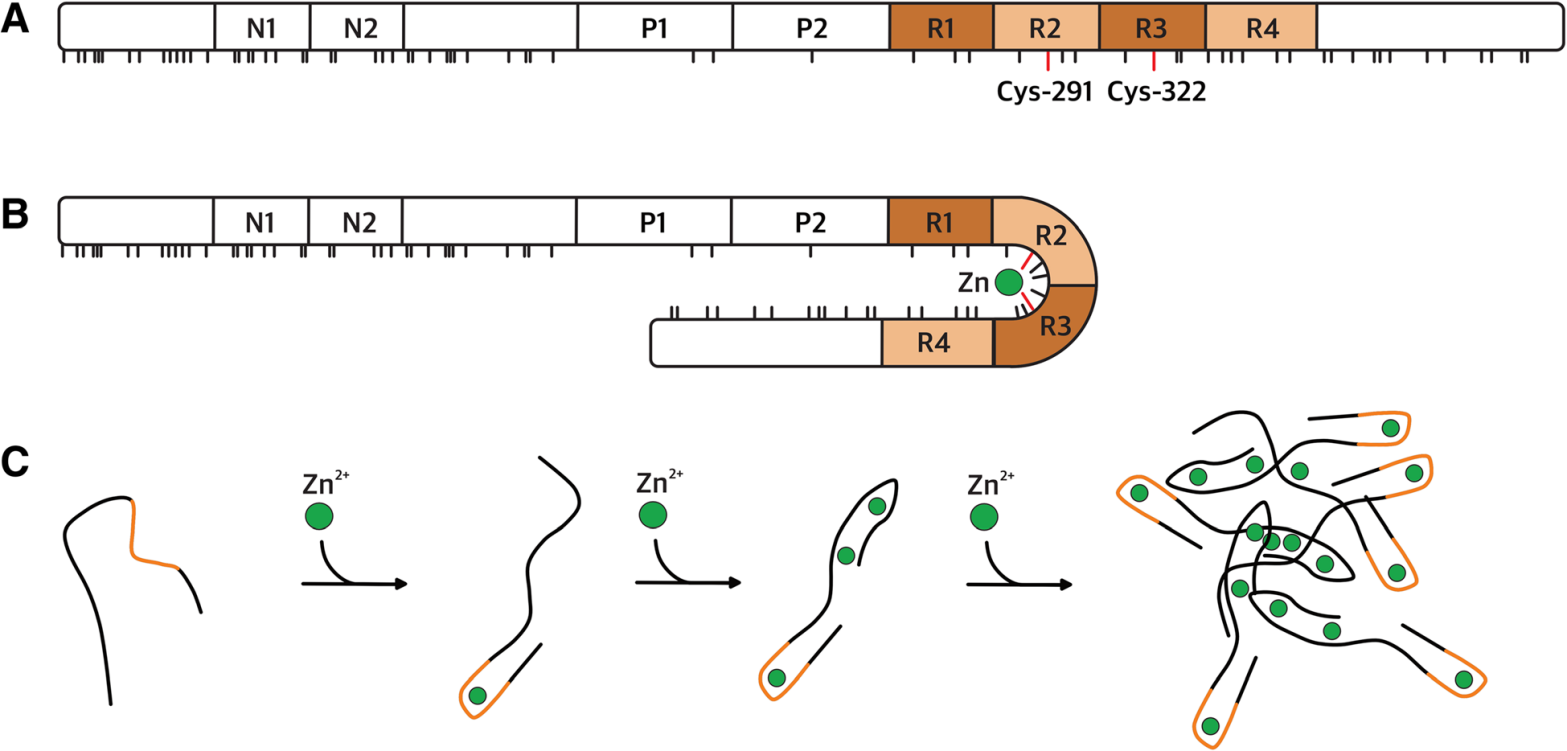
**a** Domain structure of tau2N4R with the location of potential zinc chelators shown in sticks (Cys, His, Asp, Glu). **b** Scheme of zinc chelation by the main binding sites located in R2 and R3 domains. **c** Hypothetical scheme of reversible zinc-induced aggregation

In physiological processes, zinc ions are usually involved in maintaining structure and function of hundreds of proteins, including enzymes of all known classes, transcription factors, receptors, and signalling proteins. It has even been reported that zinc was able to induce some level of structure formation in intrinsically disordered tau protein (Fig. 3). Indeed, CD spectra of tau0N4R isoform showed that zinc acts as a strong promoter of protein conformational changes [65]. Further analysis of these CD spectra using CAPTO tool [164] revealed significant increase in β-sheet content upon zinc binding, from 1% in the absence of zinc ions to 5 and 18% in the presence of 0.25 μM and 0.5 μM, respectively. This is also in-line with dynamic light scattering data presented at EuroTau meeting 2018 obtained on tau2N4R, which demonstrate a reduction in tau hydrodynamic radius in solution upon zinc binding, from 12.2 to 8.8 nm [127]. Such tau compaction and gain of structure may be explained by location of amino acids implicated in zinc chelation. Indeed, zinc ions are chelated by Cys291, Cys322 and probably by His330, His362 as shown using tau (244–372) fragment and its mutants [102]. Chelation by these residues, located in R2-R3 repeats, pulls together distant regions of tau and induces its altered conformation (Fig. 3). This was confirmed by performing Isothermal Titration Calorimetry (ITC) to monitor zinc binding to tau fragments with mutated cysteine and histidine residues. The experiments revealed a stoichiometry close to 0.5, indicative of tau fragment dimerization and of the absence of intramolecular chelation of zinc. Another study based on ITC measurements on Zn binding to tau2N4R or its cysteine mutants [64] suggested additional zinc binding sites. ITC analysis of tau2N4R interaction with zinc, presented at Eurotau 2018, confirmed the existence of one high (*N* = 1.0 ± 0.1; Ka = 2.0 ± 0.5 × 10^6^ M^− 1^) and of three low affinity binding sites (*N* = 3.2 ± 0.3; Ka = 5.9 ± 1.7 × 10^4^ M^− 1^) [127]. The high affinity site most probably corresponds to the one described previously [102] in tau (244–372) fragment, formed by two cysteines and two histidines from R2 and R3 domains (Fig. 3). Since the three auxiliary sites were not detected in tau (244–372) fragment, they could be located in N- and/or C- terminal regions, which have many potential zinc chelating amino acids. Even if we now have evidence regarding the specific tau regions implicated in zinc binding, the impact of zinc binding on tau physiological functions (including binding to tubulin) remains poorly understood. A recent study showing that zinc ions binding to tau affects its interaction with DNA offers a first step towards a better understanding of the functional aspects of Zn-binding [14].

While in many cases zinc acts as an integral component of the protein structure, it is also known for its ability to destabilize the structure of a number of proteins (e.g. NCS-1, TDP-43) [47, 151]. If not the causative agent, zinc is found implicated in the development of proteinopathies as a factor favouring aggregation. An impact of zinc binding on tau aggregation was previously shown in vivo, in cells and in vitro. Indeed, recent studies demonstrated that zinc exacerbates tau pathology in a mouse model [28] and directly regulates tau toxicity in Drosophila tauopathy model [65]. Moreover, it was shown that high concentration of zinc dramatically accelerates aggregation of full-length human tau and increases its toxicity in neuronal cells [64]. Several studies have addressed the question of whether zinc impacts on tau aggregation leading to PHF formation in vitro [64, 65, 102], yet it should be noted that they were carried out in the presence of artificial aggregation inductors such as heparin or congo red. Recently it was shown that these inducers that are classically used to stimulate PHF formation actually lead to filaments that have a different structure from those found in vivo [43]. On the contrary, turbidimetry results presented in EuroTau2018 showed that in the absence of heparin, zinc is able to induce a temperature-dependent reversible oligomerization of tau [127]. The obtained amorphous oligomers were not amyloid-like (ThT negative and no aggregates are observed by EM), and dissociated instantly following zinc chelation or a temperature decrease. At this stage it is not clear whether this newly identified Zn-induced oligomerization mechanism is part of the early stages that may lead to PHF formation, or it may be part of a concurrent pathway. In any case, a better understanding of this process at the molecular level and the role it plays in cell should not be overlooked when searching for new strategies to fight neurodegenerative diseases.

### Of mice and men

Age-related neurodegenerative disorders, including AD are largely human-specific pathologies. As a matter of fact, the human brain seems particularly susceptible to develop tau pathology leading to neurodegeneration [67]. Transgenic mice are frequently used as animal models for studying tauopathies and AD despite the fact that they do not readily develop the full set of neuropathological and/or clinical phenotypic features observed in the human pathology [96]. Nevertheless, although far from perfect they have been very useful in dissecting specific molecular pathways involved in these pathologies as well as for the preclinical evaluation of potential therapeutic agents. Most of these murine models require overexpression of human wild-type or mutant tau in order to develop significant tau pathology. Wild-type mice do not develop tau fibrillary pathology but mouse tau can be recruited in a small proportion into aggregates formed in human tau-overexpressing transgenic brains [9].

Besides the inherent anatomical and cellular differences between the human and mouse brain, one main difference between humans and mice is that the expression of tau isoforms varies in the adult brain. While adult human brain contains almost equal amounts of tau 3R and 4R isoforms, only 4R isoforms are expressed in the adult wild-type mouse brain [10]. Intriguingly, despite this divergence in tau isoform ratios between mice and humans, protein primary sequences are highly conserved between both species (88% identity for the longest tau isoform), particularly within the MTBR (98% identity) [95]. The greatest divergence, however, appears at the N-terminus where humans have a motif spanning residues 18 to 28, which is absent from the mouse tau sequence [121]. Tau amino-terminal region appears to be involved in the formation of the paper-clip conformation under physiological conditions [114].

This 18–28 primate-specific motif of unknown function has recently been reported to mediate tau interaction with a number of neuronal proteins in a species-specific manner [145]. It is worth mentioning that this motif does not include any phosphorylation sites, as the Tyr18 residue long known to be phosphorylated by Fyn tyrosine kinase to mediate tau-plasma membrane interaction [81], remains present in both human and mouse sequences. Interestingly, two closely adjacent, flanking regions to the 18–28 motif in human tau have been described to interact with plasma membrane-binding annexins and thus modulate tau subcellular localization [48].

Prion-like propagation of tau pathology involves the release of tau molecules into the extracellular space, uptake by neighboring cells and seeded aggregation of soluble proteins. Long believed to be a consequence of neuronal death, extracellular tau released from healthy neurons appears however to be a physiological process that might be disrupted in diseased brain [97]. Thus, there is increasing evidence to strongly suggest the involvement of extracellular tau species as the main agent in the propagation of neurofibrillary lesions and spreading of tau toxicity throughout different brain regions in these disorders [52, 107]. On the other hand, a growing body of evidence has accumulated in recent years to demonstrate a crucial role for the amino-terminus in tau aggregation, spreading, dendritic localization and signaling [84]. Actually, overexpression of tau in neuronal and non-neuronal cells leads to increased tau levels in the extracellular medium (reviewed in [118]). Furthermore, the various tau isoforms show different rates of tau release, whereby the N-terminal region appears to contribute to tau release from the cell to the extracellular space [31, 74, 96].

More recently, a full-length human tau lacking the 18–28 motif in the N-terminal region of human tau mentioned above (tau2N4R-Δ18–28) has been shown to be less efficiently secreted compared with wild-type tau when overexpressed in neuronal and non-neuronal cultured cells [132]. In addition, affinity chromatography analysis looking for proteins specifically interacting with that particular human tau motif identified End Binding (EB) proteins (members of the MTs plus-end tracking protein family) as binding partners. Moreover, while overexpression of EB proteins leads to an increase of tau secretion, EBs downregulation using siRNAs reduced human tau release. The authors suggest a possible crosstalk between tau and EB proteins in distal axons in neurons in which tau would contribute to proper EBs subcellular localization while EBs might favor tau release outside the cell.

Thus, the presence of the human-specific 18–28 motif at the tau N-terminal region might facilitate tau secretion and further propagation of pathology. Studying the underlying mechanisms of tau release will provide further insight into its function in physiological and pathological conditions that may lead to the identification of relevant therapeutic targets and the development of novel therapeutic agents for these devastating disorders.

### A novel panel of tau monoclonal antibodies, rendering new insights in tau structure and fluid diagnosis

A N-terminal truncated fragment of tau, broadly defined as the N-terminal part of tau combined with the proline-rich region and devoid of the repeat domain and C-terminus [16, 99, 130], is now a well-established biomarker that aids in the current set-up of new clinical criteria for AD diagnosis. Advances in MS, such as FLEXITau [88], demonstrate that tau isoforms, defined by specific PTMs, might also help in the development of tau isoform-specific assays for tauopathies [144]. Furthermore, the recent identification of seed-competent soluble tau monomers [101] may lead to tau conformation-specific assays that would be instrumental in tau-specific targeted therapy developments. Thus, set-up of tau molecular assays of antigen-antibody interactions will be essential to advance the use of tau both as fluid marker, and as therapeutic target, in major tauopathies.

Several tau immuno-assays, such as Innotest, Roche Elecsys, Fujirebio Lumipulse, MSD and EuroImmun enzyme-linked immunosorbent assay (ELISA) are currently subjected to harmonization efforts and commutability studies [11]. The EuroImmun ELISA is based on monoclonal tau antibodies raised against tau2N4R expressed in humanized yeast models [128]. The clinical value of this assay has been demonstrated in several studies [34, 147] and the monoclonal antibodies are also used to explore the potential interest of detecting tau circulating in plasma [94, 119]. Equal amounts of 3- and 4-repeat tau are found in cerebral cortex, while expression of tau is roughly two times higher in gray matter compared to white matter and cerebellum. Because assembly of tau is concentration dependent, regional variation in expression could favor its assembly. In cerebrospinal fluid (CSF), 3- and 4-repeat tau are only a minor fraction of the total protein content and thus isoform-specific immuno-assays require ultra-sensitive technology, such as immuno-PCR. Such assays could potentially aid in the differentiation of 4-repeat tauopathies from other tauopathies [87]. In a renewed effort to isolate conformational tau antibodies, an antibody with a high affinity for exon 3 (the insert N2, Fig. 3) was isolated, named 18F12. While the potential pathological role of N2-containing tau is still subjected to preclinical scientific research [84, 172], the absence of N2-containing tau in the 4-repeat specific tauopathy, argyrophilic grain disease (AGD) [124], suggests that N2-specific tau ELISA for CSF might be able to differentiate AGD from other tauopathies.

Peptide scanning demonstrates that a major determinant of the 18F12 epitope lies in tau insert N1 (Fig. 3). While Western-blot and ELISA results demonstrate an exquisite specificity of 18F12 for N2-specific tau isoforms, peptide mapping (18-mers with an overlap of 16 amino acids) have shown a major antigenic determinant of the 18F12 lies in the C-terminus of N1 (and not in N2). This epitope overlaps with the recently identified epitope of a similar high-affinity antibody, PT18. PT18’s epitope was defined as the three last amino acids of N1 and five amino acids of N2 insert in an independent characterization of N2-specific monoclonal antibodies [153], using a slightly modified approach of peptide mapping. Thus N2-specific antibodies most likely require a specific conformation of the N1-N2 junction for optimal recognition of N2 tau isoforms. While further work is needed to understand the conformational aspect of the 18F12 epitope, the fact that exon 3 expression is always associated with exon 2 presence supports a conformational affinity aspect. Since the monoclonal antibody 18F12 had a high affinity, a simple tau ELISA was built based on 18F12 as coating antibody and a N-terminal tau antibody, ADx204, allowing detection of N2-specific tau in CSF. A clinical study in several clinical groups of tauopathies, including AGD, is underway.

Tau is a protein with many PTMs and while all methods to quantify tau have their biases and limitations, widely used sandwich immunoassays are defined by the epitopes of the capturing and detector antibodies used in the assay. Therefore, as our data illustrate, a more precise description of tau antibodies used in diagnostic assays is needed and several studies suggest that this is feasible [27, 89, 136, 169]. Additionally tau protein is not only present as a soluble full-length protein [130], but also as truncated and oligomeric/fibrillar forms. Thus immuno-assays measuring these latter forms should consider epitopes specific for the fragments and target exposed epitopes in case of specific conformations because some epitopes might be buried due to a particular conformation.

To define the added clinical value of novel specific tau immuno-assays with a specific context-of-use, e.g. differentiation of tauopathies, comparing established tau immuno-assays with the novel tau assay will be needed. Finally, depending on the specificity of the novel tau antibodies (e.g. conformational or PTM-dependent), sensitive MS, such as described above (FLEXITau [88], XL-MS [101]), will be needed to validate the specificity of antibodies and assays for its targeted conformation or PTM. Combining technological advances with specific clinical cohort (context-of-use) studies [108, 117] indeed has recently led to exploring the amyloid ratio in plasma as potential surrogate for amyloid Aβ-deposition in preclinical stages of AD.

### Imaging of tau aggregates

Until recently, post-mortem examination of brain tissues was the only means available for the direct evaluation of the changes occurring in the brain of AD and non-AD tauopathy patients. Thanks to molecular neuroimaging techniques such as positron emission tomography (PET) applied with specific radiopharmaceuticals for PHFs (see [134] for a recent review), tau pathology can now be detected, characterized and quantified in the living human brain [26].

Considering the first-generation tau PET ligands, the first fluorine-18 (18F) tracer with tau binding capacity was 18F-FDDNP. However, the compound also binds to β-amyloid and suffered from a lack of selectivity [77]. Meanwhile, more-selective tracers have become available. Carbon-11 (11C) PBB3, allows tau imaging in AD and non-AD tauopathies such as corticobasal syndrome. However, the 11C label is less ideal, as it limits widespread use due to its short half-life of 20 min [137]. Other tau tracers were recently developed such as F18-AV1451 (aka F18-T807, Flortaucipir; Avid Radiopharmaceuticals) or THK5351. F18-AV1451 has demonstrated promising results and showed increased tau binding in AD. Early ex-vivo work demonstrated that F18-AV1451 selectively binds to tangles in post-mortem AD brain tissue [92]. Coupled with PET, F18-AV1451 binding is higher in patients with AD dementia or with mild cognitive impairment than in clinically normal older adults [72]. The first direct comparison of post-mortem tau pathology with in vivo regional F18-AV1451 uptake has just been published [140]. This study is based on a single patient, a man with early onset AD caused by a presenilin mutation. Results showed that in vivo tau tracer retention strongly correlated with both neuritic and intrasomal tau pathology and total tau burden, but not with amyloid plaques, at autopsy.

**Fig. 4:**
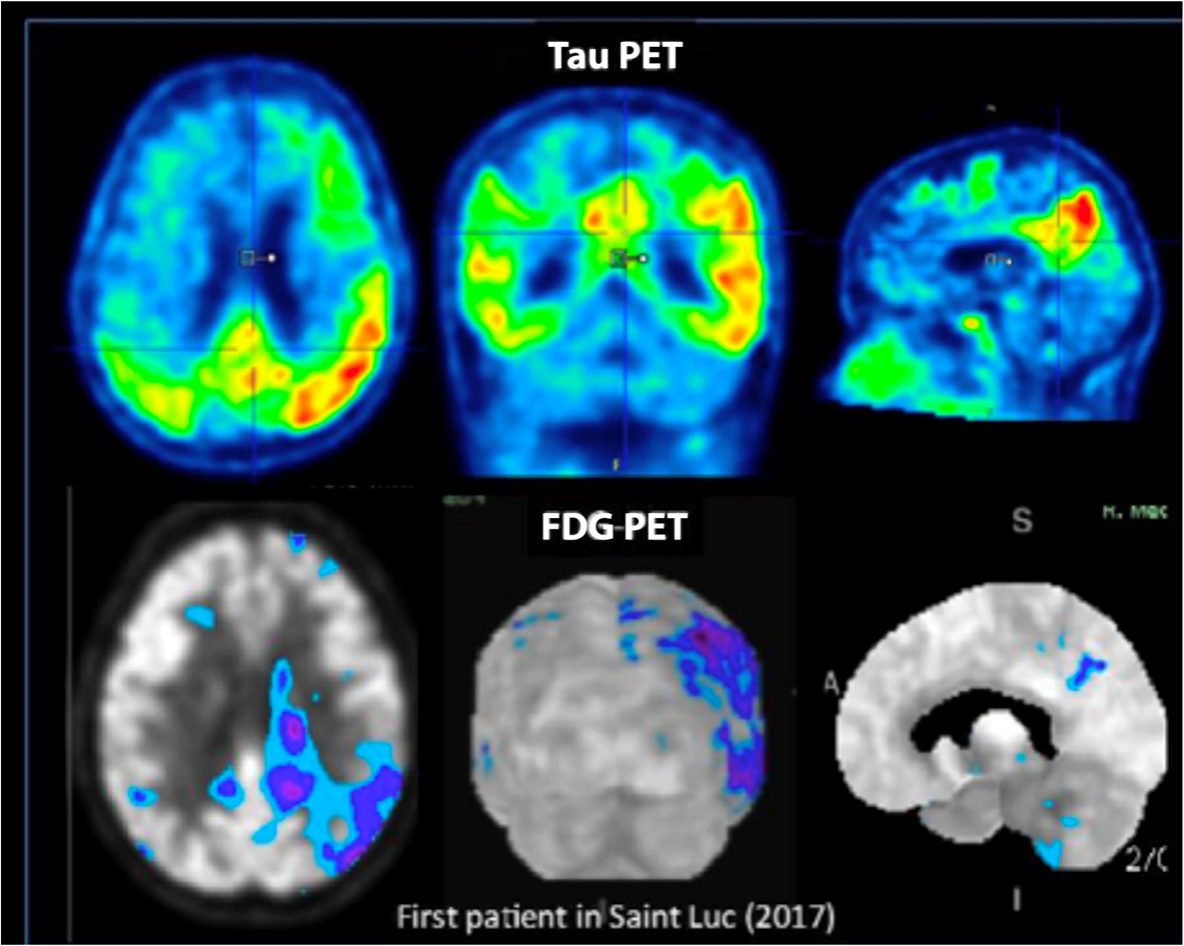
Tau PET image in a patient with AD ( Mini Mental State Examination= 20/30), demonstrating close association between tau pathology (top) and cerebral glucose metabolism (FDG-PET, bottom). Images were acquired at Saint-Luc University Hospital (UCLouvain, Belgium)

As expected from neuropathological data [109], tau-PET signal better predicts brain and cognitive dysfunctions than amyloid-PET [120] and the regional distribution of tauopathy closely matches the brain hypometabolism observed using Fluorodeoxyglucose PET [57, 116] (Fig. 4). Tau-PET signal also closely correlates with total tau and 181p-tau concentrations in the CSF [25, 78]. Given the close relationship between tau deposition, impaired cognition and neuronal injury, tau-PET is able to provide significant additive information to clinical diagnosis and amyloid-PET imaging and offers a complementary tool to help in discriminating between different pathologies, and possibly, between different tauopathies. Besides offering in-vivo images, the ability to image the presence and spatial extent of tau deposition also opens the possibility of tracking the progression of tau pathology over time [68] and detecting early changes in cognitively unimpaired individuals [59, 61]. In this respect, it has the potential to serve as a biomarker for disease severity or neurodegeneration. Moreover, the development and efficient usage of tau-directed therapeutics will be highly dependent on the presence or absence of tau and on means to identify those patients best suited for the therapy, so the utility of such disease-modifying drugs are dependent on early and accurate detection of tau. Tau-PET represents as well a non-invasive method to evaluate the efficacy of treatments with the potential to reduce tau load.

At EuroTau 2018, longitudinal tau-PET data from the Harvard Aging Brain study were presented [30, 58]. In clinically normal older individuals, changes in tau-PET signal were shown to correlate with cognitive decline. While an association between change in amyloid and change in tau was also observed, the direct relation between change in amyloid and change in cognition was rather weak [61]. The data presented advocate for sequential changes in preclinical AD from amyloidosis to tauopathy to cognitive deficits. This proof-of-concept study demonstrates the feasibility of tracking tau pathology in normal adults.

Limitations were however reported for some of these investigational first generation tau PET imaging agents concerning off-target binding in several brain regions, such as basal ganglia or choroid plexus. Particularly, off-target binding to Monoamine oxidase A (MAO-A) has been described for F18-AV1451 [155] or to MAO-B for THK5351 [110]. The presence of monoamine oxidases within several brain regions limits the interpretation of PET imaging results with these tracers. MAO-A is mostly only expressed in basal ganglia but MAO-B is expressed in cortex [110]. F18-AV1451 also suffers from off-target binding on neuromelanin present in the choroid plexus of the temporal horn of the lateral ventricles, which makes it difficult to evaluate hippocampal signal properly [80].

The second-generation of tau PET ligands is under development with the objective of breaking the limits of previously reported tau PET agents. These novel second-generation tau tracers currently investigated clinically include F18-RO6958948 (Roche), F18-GTP1 (Genentech), F18-MK-6240 (Merck/Cerveau) and F18-PI-2620 (Life Molecular Imaging). F18-PI-2620 data were presented at EuroTau 2018.

F18-PI-2620 was discovered in a research collaboration between Piramal Imaging (now Life Molecular Imaging) and AC Immune SA. Preclinical pharmacological studies indicate specific binding of F18-PI-2620 to pathological tau [146]. F18-PI-2620 shows high affinity for aggregated tau in AD brain homogenate competition-assays and PHF preparations. Autoradiography studies using human brain sections showed specific binding of F18-PI-2620 in autopsy-confirmed AD tissue sections from Braak stages I to VI, as well as to tau deposits in PSP brain tissue. F18-PI-2620 binds to both tau isoforms 3R and 4R and demonstrates high selectivity over β-amyloid, α-synuclein, MAO-A and MAO-B. F18-PI-2620 also showed low off-target binding in competition assays and autoradiography studies using brain tissue from non-demented controls. Moreover, in microPET imaging studies in mice and non-human primates, F18-PI-2620 showed high brain uptake and fast wash-out.

**Fig. 5:**
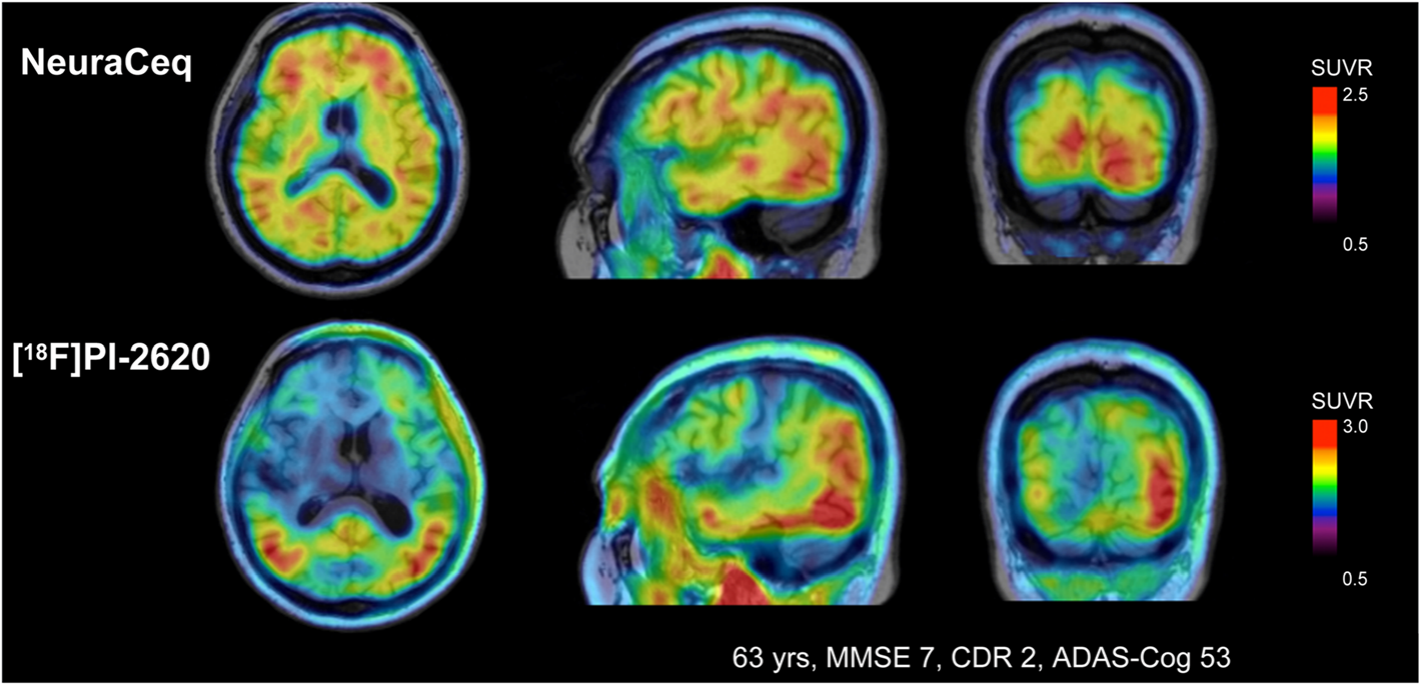
Comparison of tracer uptake patterns of F18-PI-2620 targeting tau and Neuraceq targeting β-amyloid plaques

Based on the available promising preclinical data of F18-PI-2620 the ex-vivo studies have been extended to first in-human evaluations [146]. In AD subjects, PET images of F18-PI-2620 showed a tau distribution pattern expected from typical histopathology tau-spread [22]. In contrast to the uptake seen with the β-amyloid PET tracer NeuraCeq, F18-PI-2620 showed an asymmetric uptake pattern in the temporal lobes, sparring of the motor cortex and asymmetric uptake in the frontal lobe (Fig. 5). Highest signals were observed in the temporal cortex, extending into the frontal cortex in the most severe cases. Standard uptake value ratio (SUVr) time curves suggested a plateau of the signal occurring 60–90 min post-injection with resultant SUVrs in abnormal regions up to four. Clinical data in non-demented control subjects showed robust initial brain uptake and fast wash-out from the brain. F18-PI-2620 did not exhibit an increased tracer uptake in choroid plexus, striatum, amygdala or other regions of non-demented control subjects as seen with first generation tau tracers, as well as no age dependency. Non-invasive quantification of PI-F18-PI-2620 uptake (SUVr at 60–90 min post injection) provided significant discrimination between non-demented control and AD subjects. AD subjects showed significantly higher uptake than in non-demented control subjects in temporal lobe, parietal and cingulate cortex. Moreover, excellent test-retest variability has been demonstrated and confirmed the utility of F18-PI-2620 to evaluate change of tau deposition in longitudinal studies.

Given the role of tau protein in the pathology of AD and other non-AD tauopathies, neuroimaging tau PET biomarker offers the potential to improve our understanding of the pathological process in AD and other tauopathies. Beyond the major progresses that such imaging tool offers for research on these pathologies, the ability to image tau in in vivo brain brings relevant clinical perspectives.

PET imaging appears as a useful tool for detecting the presence and spatial extent of tau deposition in in-vivo brains and offers the potential to improve our understanding of the molecular mechanism of neurofibrillary degeneration, to predict and track disease progression and to accelerate the development of rational therapies for AD and non-AD tauopathies.

## Conclusions

Recently, significant advances have been made in understanding tau structure and aggregation. The recent interest in immuno-therapies targeted against tau [69] require a good definition of what should be targeted by the assayed antibodies. Many disappointing trials could probably be avoided from a better definition of tau structure and related (dys)functions. Similarly, to be able to relate tau detection to a specific form is essential. Many studies still rely on the good old AT8 antibody to assess the level of tau pathology in cells or tissues. In this regard, imaging has also offered impressive progress that will be critical to assess any results coming from therapeutic intervention on the tau pathway. In this field, it will also be crucial to know what is traced.

While instrumentation advancements have allowed recent breakthroughs in structural biology of amyloids, there are still major fundamental challenges to solve. For example, what are the molecular factors that determine the convergence toward a given strain/structure? How can one reproduce the brain-derived filament structures with recombinant protein? While cryoEM has shown that a given tauopathy is characterized by a specific aggregate structure, it remains to be shown that a given structure is sufficient to trigger a specific disease. In other words, do the tau aggregate structures carry all the pathological information, as the prion denomination suggests, or are there other systemic factors required?

In addition, the causal relationship between tau hyperphosphorylation and aggregation in neuropathology remains to be demonstrated and to date, there are only a limited number of studies that have explored this relationship. A better definition of the pattern(s) of tau phosphorylation that might affect its conformation and lead to the aggregation path is worth the efforts, as it could help both diagnostic and therapeutic development by defining the species of tau that could be considered as targets for immune-detection or immune-intervention.

All these questions can only be addressed by bringing together the various approaches, such as NMR for flexible Tau forms and Cryo-EM for the most rigid forms or fluid immuno-diagnosis coupled to brain imaging research. The EuroTau meeting will continue to provide such opportunities.

## Abbreviations

1 N: First insert
11C: Carbon-11
18F: Fluorine-18
2 N: Second insert
3R: Three repeat
4R: Four repeat
AD: Alzheimer’s disease
AGD: Argyrophilic grain disease
CD: Circular dichroïsm
cryo-EM: Cryo-electron microscopy
CSF: Cerebrospinal fluid
EB protein: End-binding protein
ELISA: Enzyme-linked immunosorbent assay
EM: Transmission electron microscopy
EPR spectroscopy: Electron paramagnetic spectroscopy
IDP: Intrinsically disordered protein
ITC: Isothermal titration calorimetry
MRI: Magnetic resonance imaging;
MS: Mass spectrometry
MTBR: Microtubule binding region
MTs: Microtubules
NFTs: Neurofibrillary tangles
NMR: Nuclear Magnetic resonance Spectroscopy
PET: Positron emission tomography
PHF6(*): PHF6* (_275_VQIINK_280_) and PHF6 (_306_VQIVYK_311_) hexa-peptide sequences
PHFs: Paired-helical filaments
PiD: Pick’s disease
pSer/pThr: Phospho-serine/phospho-threonine
PSP: Progressive Supranuclear Palsy
PTMs: Post translational modifications
SFs: Straight Filaments
ssNMR: Solid-state NMR
SUVr: Standard uptake value ratio
ThT: ThioflavinT
